# Comparison of outcomes between preoperative and postoperative systemic treatment in patients with hepatocellular carcinoma: a SEER database-based study

**DOI:** 10.3389/fonc.2024.1324392

**Published:** 2024-03-19

**Authors:** Yadi Liu, Shuangshuang Sun, Zhaoyin Chu, Caixia Liu, Lina Chen, Zhengshang Ruan

**Affiliations:** ^1^ Xinhua Hospital, School of Medicine, Shanghai Jiao Tong University, Shanghai, China; ^2^ Department of Liver Disease, Shanghai Public Health Clinical Center, Fudan University, Shanghai, China

**Keywords:** hepatocellular carcinoma(HCC), preoperative systemic treatment, postoperative systemic treatment, overall survival(OS), cancer-specific survival(CSS), SEER database

## Abstract

**Background:**

Significant advancements in systemic treatment for hepatocellular carcinoma have been made in recent years. However, the optimal timing of systemic treatment before or after surgery remains unknown. This study aims to evaluate the impact of sequencing systemic treatment and surgical intervention on the long-term prognosis of hepatocellular carcinoma patients.

**Methods:**

In our study, we analyzed data from patients diagnosed with primary liver cancer (2004-2015) extracted from the SEER database. Patients who underwent both systemic treatment and surgical intervention were selected, divided into preoperative and postoperative systemic therapy groups. The primary endpoint of the study is overall survival(OS), and the secondary endpoint is cancer-specific survival (CSS). Propensity score matching (PSM) reduced the influence of confounding factors, while Kaplan-Meier curves and a multivariable Cox proportional hazards model accounted for variables during survival analysis.

**Results:**

A total of 1918 eligible HCC patients were included, with 1406 cases in the preoperative systemic treatment group and 512 cases in the postoperative systemic treatment group. Survival analysis showed that both the preoperative group demonstrated longer median overall survival (OS) and median cancer-specific survival (CSS) before and after PSM. After conducting multivariate COX regression analysis with stepwise adjustment of input variables, the postoperative systemic treatment group continued to exhibit a higher risk of all-cause mortality (HR: 1.84, 95% CI: 1.55-2.1) and cancer-specific mortality (HR: 2.10, 95% CI: 1.73-2.54). Subgroup analysis indicated consistent results for overall survival (OS) across different subgroups.

**Conclusions:**

Hepatocellular carcinoma patients from the SEER database who received preoperative systemic therapy had superior OS and CSS compared to those who received postoperative systemic therapy.

## 1 Introduction

Based on 2020 global cancer statistics, primary liver cancer ranks as the sixth most common cancer and the third leading cause of cancer-related deaths worldwide. Approximately 906,000 new cases are diagnosed annually, resulting in 830,000 deaths. Liver cancer holds the fifth position in terms of global incidence rates and ranks second in male mortality rates. In primary liver cancer, 75-85% of cases are attributed to Hepatocellular carcinoma (HCC) (1). Despite the rising incidence of HCC worldwide, significant progress has been achieved in HCC treatment in recent years. Particularly, the development of novel systemic treatment approaches has notably improved overall survival rates and quality of life (2). The treatment for early-stage HCC involves three strategies: tumor resection, local percutaneous ablation, and liver transplantation, with 5-year survival rates ranging from approximately 70% to 80%. For intermediate-stage HCC, patients can choose between liver transplantation, transarterial chemoembolization (TACE), and systemic treatment based on tumor burden and liver function. In the case of advanced-stage HCC, systemic treatment becomes the primary mode of therapy (3, 4).

Surgical intervention is the preferred and crucial treatment for early-stage liver cancer, and it can also be considered for select patients with advanced-stage cancer, significantly improving long-term survival rates. However, the benefits of surgical treatment for liver cancer patients are currently constrained in two areas. Firstly, although surgical resection is not the primary approach for intermediate or advanced-stage liver cancer, there are still suitable candidates in these stages who can benefit from surgery. Expanding the criteria for surgical resection to encompass more patients in the intermediate and advanced stages is an urgent concern. Secondly, the postoperative recurrence rate remains relatively high for liver cancer patients, even among those with early-stage cancer, with an approximate 60% recurrence rate within five years after surgery (5). Patients with advanced-stage HCC have a higher postoperative recurrence rate compared to those in the early-stage. Addressing the reduction of postoperative recurrence rate is thus a crucial issue to tackle.

With the advancements in molecular targeted therapy and immunotherapy, targeted immunotherapy-based systemic treatment is gradually infiltrating perioperative care (6). The combination of systemic treatment and surgical intervention can enhance the effectiveness of liver cancer treatment (7). Some studies suggest that preoperative systemic treatment can reduce tumor volume and improve the success rate of surgical resection (8). However, other research indicates that postoperative systemic treatment can eliminate residual tumor cells, lower tumor recurrence rates, and prolong overall survival (9). Given the diverse treatment options available for liver cancer, both preoperative and postoperative systemic treatments are widely employed. However, there remains controversy regarding the comparison of long-term survival outcomes between these two treatment modalities in hepatocellular carcinoma patients. Consequently, more research is needed to aid clinical decision-making regarding the selection of preoperative or postoperative systemic therapy for HCC patients.

This study seeks to assess and compare the efficacy of preoperative and postoperative systemic treatment in managing HCC, while investigating their influence on long-term survival rates. It will provide valuable guidance for the selection of treatment strategies for HCC patients, thereby improving treatment outcomes and long-term prognosis.

## 2 Materials and methods

### 2.1 Patient selection

**Figure 1:**
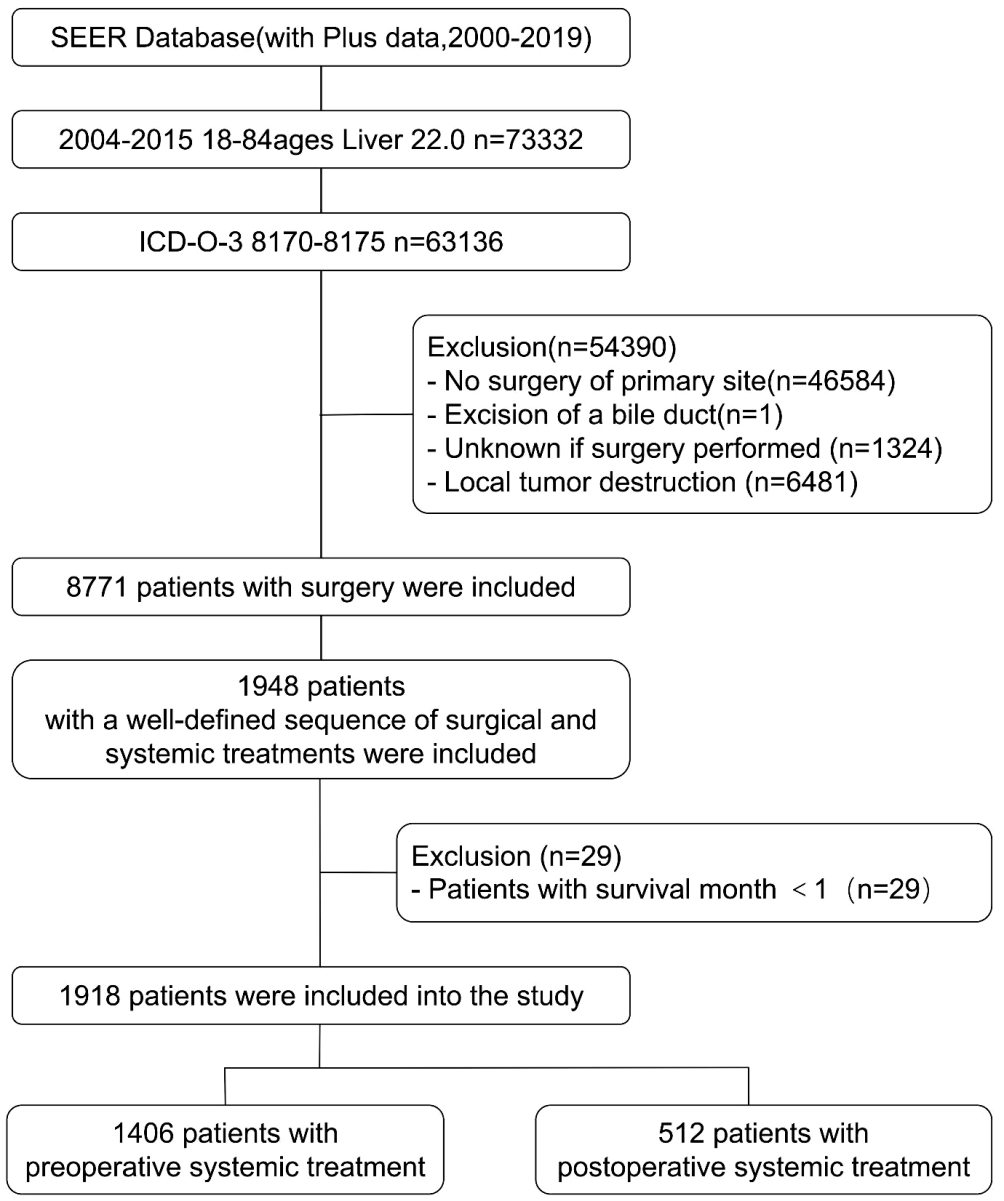
The flowchart for patient selection from the SEER database for comparing outcomes between preoperative and postoperative systematic treatment.

The SEER (Surveillance, Epidemiology, and End Results) database, maintained by the National Cancer Institute under the National Institutes of Health in the United States, is a comprehensive cancer registry covering approximately 28% of the U.S. population. It collects cancer case data dating back to 1973, including information on patients’ age, gender, race, tumor site, staging, treatment modalities, and follow-up information. In this study, we utilized the SEER*STAT software (version 8.4.0.1) to query and download clinical, treatment and follow-up data of HCC patients diagnosed between 2004 and 2015. The inclusion criteria for this study were as follows: age range of 18-84 years old, site code is “C22.0”, ICD-O-3 histology/behavior codes for malignant tumors is “8170-8175”, and year of diagnosis is between 2004 and 2015. A total of 63,161 patients were selected. Patients with incomplete data or a survival period of less than one month were excluded from the study. The selection criteria for patients are shown in **Figure 1**. After selection, a total of 1918 patients with a well-defined sequence of surgical and systemic treatments were included. Based on the order of surgery and systemic treatment, they were divided into two groups: the preoperative systemic treatment group, referring to patients who underwent systemic treatment before surgery, including 1406 individuals; and the postoperative systemic treatment group, referring to patients who underwent surgery first and then received systemic treatment, including 512 individuals.

### 2.2 Variables and endpoints

Basic demographic information includes gender, age, race, and marital status. Clinical and pathological data encompass the year of diagnosis, the 7th edition of the American Joint Committee on Cancer (AJCC) staging system, AJCC N stage, AJCC T stage, AJCC M stage, tumor size, and tumor count. Treatment information includes chemotherapy, radiotherapy, surgical methods, as well as the sequence of systemic treatment and surgery. In the SEER database, only patients included between 2010 and 2015 were staged using the 7th edition of the AJCC staging system. To ensure consistent staging criteria for all eligible patients, we employed a combination of variables, including CS Extension, CS Lymph Nodes, CS Mets at DX, and CS tumor size, to determine the 7th edition AJCC staging for other patients. The study outcomes of interest encompass overall survival (OS) and cancer-specific survival (CSS). Overall survival refers to the time from the diagnosis to death from any cause. Cancer-specific survival, on the other hand, refers to the time from the diagnosis to death specifically attributed to cancer.

### 2.3 Statistical analysis

In this study, we utilized the SEER*SAT 8.4.0.1 software to extract raw data and conducted categorical transformation for all continuous variables. Statistical analysis was performed using SPSS 24.0. Differences in baseline characteristics before and after PSM between the preoperative systemic treatment group and the postoperative systemic treatment group were assessed via Chi-squared tests or Fisher’s exact test when sample sizes were small. To explore the prognostic survival of both groups, Kaplan-Meier survival curves were generated using R software version 4.2.3, and the Log-rank test was employed to compare survival rates between the two groups. Additionally, forest plots for subgroup analysis were created, and interaction P-values (P for interaction) were computed to evaluate the heterogeneity of effects among groups. Throughout the study, a significance level of P < 0.05 was considered statistically significant.

To control for potential confounding factors, we employed two methods. Firstly, we balanced baseline characteristics between the preoperative and postoperative systemic treatment groups using PSM, encompassing all relevant covariates. The optimal nearest neighbor ratio for PSM was set at 0.1, achieving successful 1:1 exact matching for 353 pairs of patients.

Secondly, during the regression analysis phase, we utilized a stratified regression method to further explore the impact of each covariate on patient prognosis. Specifically, we initially applied the univariate COX proportional hazards model to evaluate the hazard ratios (HRs) and corresponding 95% confidence intervals (CIs) for OS and CSS of the two patient groups, both pre and post PSM correction. Subsequently, we constructed a multivariable COX proportional hazards model for stepwise variable adjustment: Model I included basic demographic variables such as age, gender, and race; Model II incorporated tumor-related variables including tumor count, tumor size, TNM staging, and AJCC staging information upon the basic variables; finally, in Model III, treatment-related factors such as radiation therapy and chemotherapy were further considered, forming a comprehensive model containing all study covariates. Adjusted hazard ratios (AHRs) and their corresponding 95% CIs were computed for each stratum in this complete model.

## 3 Results

### 3.1 Characteristics of patients

This study included a total of 1,918 HCC patients, including 1,459 males and 459 females. The baseline characteristics of these patients are shown in **Table 1**. Among them, 1406 received preoperative systemic treatment, and 512 received postoperative systemic treatment. In the preoperative systemic group, there were 1227 cases of AJCC stage I and II patients (87.3%), among which 258 cases (18.3%) underwent surgical resection, 1,148 cases (81.7%) underwent liver transplantation, and 1146 cases (81.5%) had a solitary tumor. In contrast, in the postoperative systemic group, there were 299 cases of AJCC stage I+II patients (58.4%), among which 411 cases (80.3%) underwent surgical resection, 101 cases (19.7%) underwent liver transplantation, and 411 cases (80.3%) had a solitary tumor. Further analysis of the relationship between surgical approach and sequence of systemic treatment revealed that among the 1249 patients with liver transplantation, 1148 cases (91.9%) received preoperative systemic treatment, while among the 669 patients with surgical resection, 411 cases (61.4%) received postoperative systemic treatment. Patients undergoing liver transplantation were more inclined to receive preoperative systemic treatment (91.9% vs 8.1%), whereas patients undergoing surgical resection were more inclined to receive postoperative systemic treatment (61.4% vs 38.6%).

**Table 1:** The baseline characteristics of patients before and after PSM.

Characteristics	Before PSM			After PSM		
	Before surgery (n=1406, %)	After surgery (n=512, %)	P value	Before surgery (n=353, %)	After surgery (n=353, %)	P value
Age (Years)			<0.001			0.332
18-44	52 (3.7)	49 (9.6)		19 (5.4)	29 (8.2)	
45-54	276 (19.6)	89 (17.4)		58 (16.4)	65 (18.4)	
55-64	760 (54.1)	203 (39.6)		164 (46.5)	147 (41.6)	
65-74	289 (20.6)	123 (24)		88 (24.9)	81 (22.9)	
≥75	29 (2.1)	48 (9.4)		24 (6.8)	31 (8.8)	
Gender			<0.001			0.302
Male	1099 (78.2)	360 (70.3)		268 (75.9)	256 (72.5)	
Female	307 (21.8)	152 (29.7)		85 (24.1)	97 (27.5)	
Race			<0.001			0.387
White	1006 (71.6)	311 (60.7)		203 (57.5)	219 (62.0)	
Black	134 (9.5)	66 (12.9)		43 (12.2)	43 (12.2)	
Other	266 (18.9)	135 (26.4)		107 (30.3)	91 (25.8)	
Marital status			0.479			
Single	406 (28.9)	161 (31.4)		118 (33.4)	104 (29.5)	0.482
Married	938 (66.7)	332 (64.8)		222 (62.9)	233 (66.0)	
Unknown	62 (4.4)	19 (3.7)		13 (3.7)	16 (4.5)	
Year of diagnosis			<0.001			0.152
2006-2010	616 (43.8)	277 (54.1)		156 (44.2)	175 (49.6)	
2011-2015	790 (56.2)	235 (45.9)		197 (55.8)	178 (50.4)	
AJCC staging			<0.001			0.126
I	662 (47.1)	157 (30.7)		157 (44.5)	133 (37.7)	
II	565 (40.2)	142 (27.7)		102 (28.9)	98 (27.8)	
III	114 (8.1)	134 (26.2)		69 (19.5)	80 (22.7)	
IV	29 (2.1)	60 (11.7)		16 (4.5)	27 (7.6)	
Unknown	36 (2.6)	19 (3.7)		9 (2.5)	15 (4.2)	
AJCC T			<0.001			0.206
T1	682 (48.5)	171 (33.4)		161 (45.6)	140 (39.7)	
T2	583 (41.5)	157 (30.7)		109 (30.9)	106 (30.0)	
T3	108 (7.7)	126 (24.6)		66 (18.7)	78 (22.1)	
T4	22 (1.6)	47 (9.2)		13 (3.7)	20 (5.7)	
Tx	11 (0.8)	11 (2.1)		4 (1.1)	9 (2.5)	
AJCC N			<0.001			0.314
N0	1355 (96.4)	463 (90.4)		334 (94.6)	324 (91.8)	
N1	20 (1.4)	27 (5.3)		10 (2.8)	14 (4.0)	
Nx	31 (2.2)	22 (4.3)		9 (2.5)	15 (4.2)	
AJCC M			<0.001			0.110
M0	1395 (99.2)	470 (91.8)		344 (97.5)	336 (95.2)	
M1	11 (0.8)	42 (8.2)		9 (2.5)	17 (4.8)	
Tumor size (cm)			<0.001			0.174
≤2	280 (19.9)	56 (10.9)		36 (10.2)	39 (11.0)	
>2, ≤5	845 (60.1)	173 (33.8)		136 (38.5)	135 (38.2)	
>5, ≤10	181 (12.9)	133 (26.0)		107 (30.3)	83 (23.5)	
>10	70 (5.0)	116 (22.7)		62 (17.6)	77 (21.8)	
Unknown	30 (2.1)	34 (6.6)		12 (3.4)	19 (5.4)	
Tumor number			0.888			0.375
1	1146 (81.5)	411 (80.3)		282 (79.9)	288 (81.6)	
2	217 (15.4)	86 (16.8)		55 (15.6)	56 (15.9)	
3	36 (2.6)	12 (2.3)		11 (3.1)	8 (2.3)	
>3	7 (0.5)	3 (0.6)		5 (1.4)	1 (0.3)	
Radiation			<0.001			0.160
No/Unknown	1357 (96.5)	468 (91.4)		340 (96.3)	332 (94.1)	
Yes	49 (3.5)	44 (8.6)		13 (3.7)	21 (5.9)	
Chemotherapy			<0.001			0.203
No	3 (0.2)	16 (3.1)		3 (0.8%)	7 (2.0)	
Yes	1403 (99.8)	496 (96.9)		350 (99.2)	346 (98.0)	
Surgery			<0.001			0.932
Liver resection	258 (18.3)	411 (80.3)		258 (73.1)	257 (72.8)	
Transplantation	1148 (81.7)	101 (19.7)		95 (26.9)	96 (27.2)	

Among all the variables included, before propensity score matching (PSM), there were no statistically significant differences in the distribution of marital status and tumor quantity between the two groups (both P > 0.05). To reduce the impact of confounding factors, the PSM method was employed. After PSM, a total of 706 patients were successfully matched. Among these matched patients, there were no statistically significant differences in baseline characteristics between the two groups (P > 0.05). To assess the balance of baseline data between the two groups after matching, standardized mean differences (SMD) were used. As shown in **Supplementary Figure S1**, after PSM, the SMD was significantly smaller than before PSM, and all SMD values were less than 0.2, with most of them around 0.1, indicating acceptable quality of variable balancing.

### 3.2 Survival analysis

**Figure 2:**
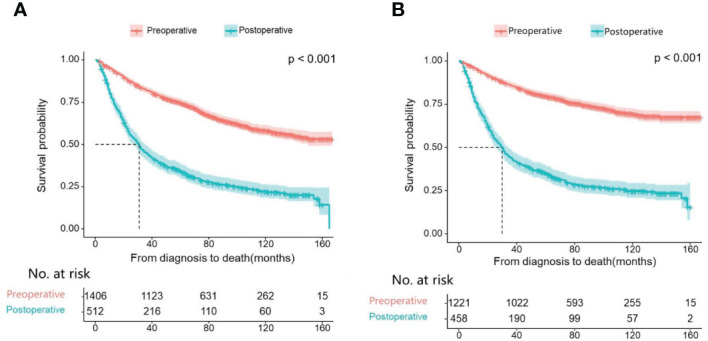
Kaplan Meier curve of patients before PSM; **(A)** Kaplan Meier curve of overall survival (OS); **(B)** Kaplan Meier curve of cancer-specific survival (CSS).

**Figure 3:**
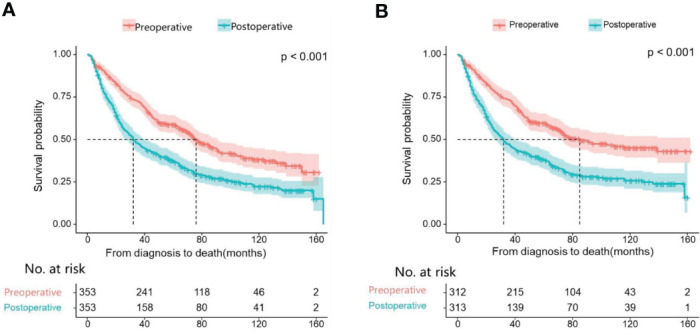
Kaplan Meier curve of patients after PSM; **(A)** Kaplan Meier curve of overall survival (OS); **(B)** Kaplan Meier curve of cancer-specific survival (CSS).

First, we compare the efficacy between the preoperative systemic treatment group and the postoperative systemic treatment group. Before PSM, the median OS and median CSS in the preoperative group were NA months (95% CI: 150–NA) and NA months (95% CI: NA–NA), respectively. These values were longer than those in the postoperative group (median OS 31 months, 95% CI: 27–35; median CSS 30months, 95% CI:26–34; both P < 0.001) (**Figure 2**). After adjusting for various covariates such as age, gender, race, and marital status, the multivariate regression analysis showed that postoperative systemic treatment patients had a 1.84 times higher risk of overall survival (HR=1.84, 95% CI: 1.55-2.17; P<0.001) compared to the preoperative group. Additionally, the postoperative systemic treatment patients had a 2.10 times higher risk of cancer-specific survival (HR=2.10, 95% CI: 1.73-2.54; P<0.001) compared to the preoperative group (**Table 2**). After PSM, the median OS and median CSS in the preoperative group were 76 months (95% CI: 70–91) and 85 months (95% CI: 72–NA),respectively. These values were longer than those in the postoperative group (median OS 32months, 95% CI: 27–41; median CSS 32months, 95% CI:26–41; both P < 0.001) (**Figure 3**). Multivariate regression analysis showed that postoperative systemic treatment patients had a 1.81 times higher risk of overall survival (HR=1.81, 95% CI: 1.49-2.20; P<0.001) compared to the preoperative group. Additionally, the postoperative systemic treatment patients had a 1.96 times higher risk of cancer-specific survival (HR=1.96, 95% CI: 1.58-2.43; P<0.001) compared to the preoperative group (**Table 3**).

**Table 2:** Cox Proportional Hazard Models for OS and CSS of patients Before PSM.

Characteristics	OS		CSS	
	HR (95%CI)	P value	HR (95%CI)	P value
Age (Years)				
18-44	Reference		Reference	
45-54	1.00 (0.72-1.39)	0.998	0.96 (0.68-1.35)	0.792
55-64	1.15 (0.85-1.56)	0.358	0.95 (0.69-1.31)	0.760
65-74	1.39 (1.01-1.91)	0.042	1.19 (0.85-1.67)	0.303
≥75	1.72 (1.17-2.53)	0.006	1.45 (0.96-2.18)	0.079
Gender				
Male	Reference		Reference	
Female	0.87 (0.74-1.02)	0.093	0.87 (0.72-1.04)	0.124
Race				
White	Reference		Reference	
Black	1.22 (0.99-1.49)	0.060	1.17 (0.92-1.49)	0.210
Other	0.79 (0.67-0.94)	0.009	0.85 (0.70-1.04)	0.112
Marital status				
Single	Reference		Reference	
Married	0.81 (0.70-0.94)	0.005	0.79 (0.66-0.93)	0.006
Unknown	0.73 (0.51-1.04)	0.081	0.62 (0.40-0.97)	0.037
Year of diagnosis				
2006-2010	Reference		Reference	
2011-2015	0.99 (0.85-1.14)	0.832	1.06 (0.89-1.25)	0.529
AJCC staging				
I	Reference		Reference	
II	0.51 (0.26-0.99)	0.048	0.56 (0.28-1.14)	0.018
III	1.10 (0.59-2.07)	0.762	1.51 (0.79-2.89)	0.216
IV	0.76 (0.30-1.97)	0.576	1.15 (0.41-3.23)	0.785
Unknown	0.46 (0.17-1.24)	0.124	1.05 (0.32-3.40)	0.940
AJCC T				
T1	Reference		Reference	
T2	2.33 (1.21-4.49)	0.011	2.37 (1.20-4.67)	0.013
T3	1.67 (0.91-3.09)	0.099	1.42 (0.76-2.66)	0.269
T4	2.03 (1.06-3.86)	0.032	1.79 (0.93-3.47)	0.082
Tx	2.05 (0.93-4.51)	0.075	1.32 (0.55-3.20)	0.528
AJCC N				
N0	Reference		Reference	
N1	1.37 (0.68-2.77)	0.379	1.32 (0.62-2.82)	0.468
Nx	1.57 (0.69-3.60)	0.283	0.98 (0.35-2.70)	0.963
AJCC M				
M0	Reference		Reference	
M1	1.62 (0.79-3.35)	0.191	1.32 (0.60-2.89)	0.489
Tumor size (cm)				
≤2	Reference		Reference	
>2, ≤5	1.23 (0.99-1.52)	0.060	1.43 (1.08-1.89)	0.012
>5, ≤10	1.27 (0.97-1.67)	0.081	1.52 (1.09-2.12)	0.013
>10	1.73 (1.29-2.32)	<0.001	2.14 (1.51-3.05)	<0.001
Unknown	1.46 (0.95-2.24)	0.082	1.62 (0.98-2.71)	0.063
Tumor number				
1	Reference		Reference	
2	1.04 (0.87-1.24)	0.699	0.80 (0.64-1.00)	0.051
3	1.29 (0.90-1.85)	0.171	0.78 (0.46-1.32)	0.350
>3	1.48 (0.73-3.01)	0.281	0.80 (0.25-2.52)	0.704
Radiation				
No/Unknown	Reference		Reference	
Yes	1.15 (0.87-1.53)	0.332	1.31 (0.97-1.78)	0.078
Chemotherapy				
No	Reference		Reference	
Yes	1.41 (0.66-3.01)	0.375	1.43 (0.59-3.50)	0.432
Surgery				
Liver resection	Reference		Reference	
Transplantation	0.54 (0.44-0.65)	<0.001	0.43 (0.35-0.54)	<0.001
Systemic Sur Seq				
Before-surgery	Reference		Reference	
After-surgery	1.84 (1.55-2.17)	<0.001	2.10 (1.73-2.54)	<0.001

**Table 3:** Cox Proportional Hazard Models for OS and CSS of patients After PSM.

Characteristics	OS		CSS	
	HR (95%CI)	P value	HR (95%CI)	HR (95%CI)
Age (Years)				
18-44	Reference		Reference	
45-54	0.97 (0.63-1.49)	0.901	0.93 (0.60-1.45)	0.760
55-64	0.83 (0.55-1.23)	0.351	0.69 (0.46-1.05)	0.081
65-74	1.00 (0.65-1.52)	0.988	0.84 (0.54-1.30)	0.430
≥75	1.31 (0.81-2.13)	0.269	1.00 (0.60-1.68)	0.992
Gender				
Male	Reference		Reference	
Female	0.70 (0.56-0.88)	0.003	0.75 (0.58-0.96)	0.021
Race				
White	Reference		Reference	
Black	1.10 (0.83-1.47)	0.510	1.18 (0.86-1.61)	0.318
Other	0.73 (0.58-0.92)	0.008	0.76 (0.59-0.96)	0.038
Marital status				
Single	Reference		Reference	
Married	0.87 (0.70-1.08)	0.200	0.85 (0.68-1.08)	0.181
Unknown	0.67 (0.40-1.12)	0.123	0.65 (0.36-1.16)	0.144
Year of diagnosis				
2006-2010	Reference		Reference	
2011-2015	1.15 (0.94-1.40)	0.173	1.12 (0.90-1.39)	0.303
AJCC staging				
I	Reference		Reference	
II	0.63 (0.25-1.57)	0.320	0.72 (0.28-1.84)	0.496
III	1.65 (0.70-3.90)	0.253	2.30 (0.95-5.55)	0.065
IV	4.28 (1.17-15.66)	0.028	6.59 (1.65-26.24)	0.007
Unknown	0.60 (0.15-2.38)	0.462	2.18 (0.34-14.00)	0.410
AJCC T				
T1	Reference		Reference	
T2	2.04 (0.84-4.94)	0.113	1.95 (0.80-4.78)	0.143
T3	0.98 (0.42-2.26)	0.953	0.78 (0.33-1.84)	0.572
T4	1.79 (0.73-4.34)	0.201	1.39 (0.56-3.46)	0.483
Tx	1.75 (0.58-5.26)	0.322	0.80 (0.22-2.92)	0.730
AJCC N				
N0	Reference		Reference	
N1	0.37 (0.14-0.98)	0.046	0.35 (0.12-1.01)	0.052
Nx	1.32 (0.37-4.69)	0.666	0.54 (0.10-2.93)	0.472
AJCC M				
M0	Reference		Reference	
M1	0.88 (0.32-2.47)	0.812	0.70 (0.23-2.10)	0.525
Tumor size (cm)				
≤2	Reference		Reference	
>2, ≤5	1.32 (0.91-1.91)	0.148	1.18 (0.79-1.78)	0.426
>5, ≤10	1.43 (0.96-2.14)	0.079	1.36 (0.88-2.11)	0.166
>10	2.06 (1.36-3.12)	0.001	2.02 (1.29-3.17)	0.002
Unknown	1.89 (1.00-3.58)	0.049	1.76 (0.86-3.68)	0.123
Tumor number				
1	Reference		Reference	
2	0.74 (0.56-0.97)	0.032	0.65 (0.47-0.83)	0.008
3	1.32 (0.78-2.25)	0.299	0.92 (0.46-1.84)	0.812
>3	1.00 (0.36-2.78)	0.996	0.37 (0.05-2.73)	0.332
Radiation				
No/Unknown	Reference		Reference	
Yes	1.07 (0.70-1.63)	0.743	1.15 (0.73-1.80)	0.546
Chemotherapy				
No	Reference		Reference	
Yes	1.62 (0.59-4.46)	0.348	2.65 (0.64-10.93)	0.177
Surgery				
Liver resection	Reference		Reference	
Transplantation	0.62 (0.48-0.80)	<0.001	0.57 (0.43-0.76)	<0.001
Systemic Sur Seq				
Before-surgery	Reference		Reference	
After-surgery	1.81 (1.49-2.20)	<0.001	1.96 (1.58-2.43)	<0.001

Then, we analyzed the relationships between OS and CSS with the included variables, as shown in **Table 4**. In the unadjusted model, patients who received postoperative systemic treatment had shorter OS (HR=3.24, 95% CI: 2.84-3.71; P<0.001) and shorter CSS (HR=4.31, 95% CI: 3.69-5.03; P<0.001), This is consistent with the analysis of the Kaplan-Meier (KM) curves. After stepwise adjustments for age, sex, and race (Model I), further adjustments for tumor size, tumor quantity, TNM stage, AJCC stage, and year of diagnosis (Model II), as well as additional adjustments for chemotherapy, radiotherapy and surgical methods on the foundation of Model II (Model III), and after conducting single-factor analysis following PSM, the significance of these relationships remained. This also demonstrates that the results are highly robust. It can be observed that with the increase in variables adjusted for influencing the outcomes, the risk ratio of the postoperative systemic treatment group relative to the preoperative group showed a decreasing trend, but it remained greater than 1 in all cases.

**Table 4:** The relationship between the sequence of treatment modalities (systemic treatment and surgery) and OS and CSS.

		Non-adjusted		Adjust I ^a^		Adjust II ^b^		Adjust III ^c^		Post-PSM	
		HR (95%CI)	P	HR (95%CI)	P	HR (95%CI)	P	HR (95%CI)	P	HR (95%CI)	P
OS	pre-surgery	reference		reference		reference		reference		reference	
	post-surgery	3.24 (2.84-3.71)	<0.001	3.06 (2.66-3.52)	<0.001	2.34 (2.01-2.72)	<0.001	1.84 (1.55-2.17)	<0.001	1.74 (1.44-2.09)	<0.001
CSS	pre-surgery	reference		reference		reference		reference		reference	
	post-surgery	4.31 (3.69-5.03)	<0.001	4.01 (3.41-4.70)	<0.001	2.90 (2.44-3.45)	<0.001	2.10 (1.73-2.54)	<0.001	1.86 (1.52-2.29)	<0.001

a. Adjust I: Adjusted basic demographic information (Gender, Martial, Race, Age).

b. Adjust II: Adjusted basic demographic and fundamental oncological information (Gender, Martial, Race, Age, Year of diagnosis, AJCC staging, AJCC T/N/M, Tumor size, Tumor number).

c. Adjust III: Adjusted basic demographic, fundamental oncological, and treatment information (including all incorporated variables).

**Figure 4:**
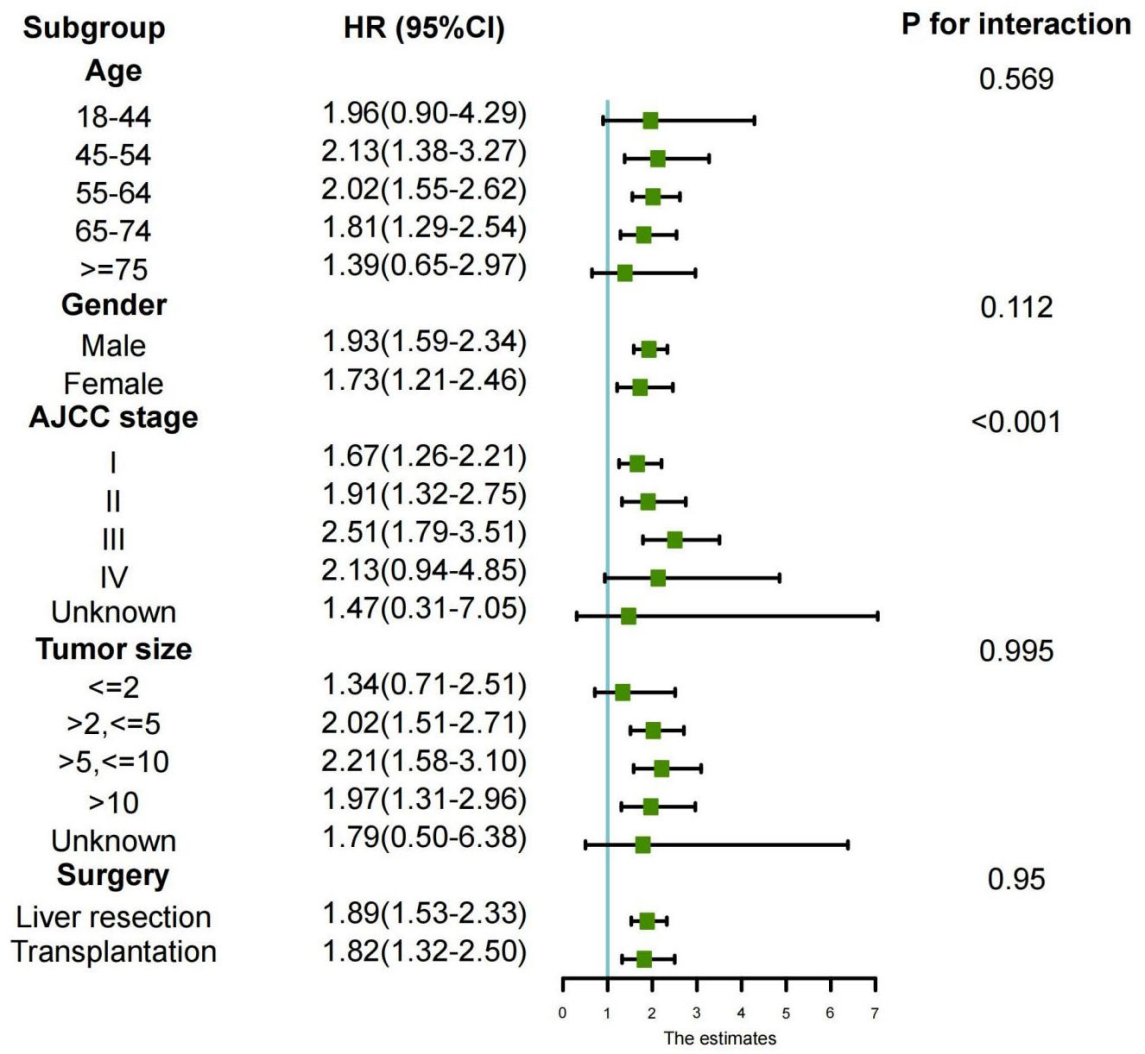
Subgroup analyses of the association between overall survival and treatment modalities according to baseline characteristics (forest plot).

Lastly, to explore the differences between different subgroups and the relationships between variables, we also conducted a subgroup analysis, as shown in **Figure 4**. The results of the subgroup analysis showed that the longer overall survival (OS) in the preoperative group was consistent with the results of the multivariate Cox regression analysis. This was particularly evident among HCC patients aged between 45 and 75 years, with AJCC stages I to III, and tumor sizes larger than 2 centimeters. Consistency of results across multiple subgroups further enhances the credibility and generalizability of our study.

## 4 Discussion

The most effective treatments for curing HCC are surgical resection and liver transplantation. Although the Barcelona Clinic Liver Cancer (BCLC) staging and guidelines only endorse surgical resection solely for early-stage HCC patients (4), there are also cases where surgical resection is deemed appropriate for intermediate and advanced HCC patients as well (10). Expanding the surgical criteria to include intermediate and advanced stage patients provides more individuals with the opportunity for surgical treatment, but it results in a significantly higher postoperative recurrence rate compared to early-stage HCC patients. Among patients undergoing surgical resection, up to 80% experience tumor recurrence (11). Even in very early-stage tumors (BCLC stage 0), the 5-year recurrence rate is approximately 60% (5). Among patients with surgical recurrence, over 70% experience recurrence within 2 years, and their prognosis is poor (12). In contrast, liver transplantation in HCC patients has a relatively lower recurrence rate, around 10-15%, typically occurring within the first year (13). Therefore, recurrence is the primary challenge affecting the long-term prognosis of postoperative patients. The main risk factors for postoperative recurrence include microvascular invasion of the primary tumor, liver cirrhosis, high AFP levels, and larger tumor volume (14, 15). Considering these factors, adopting strategies that offer more opportunities for surgical treatment and reduce the postoperative recurrence rate in hepatocellular carcinoma patients is of utmost importance.

Since the approval of Sorafenib as a first-line treatment for HCC by the FDA in 2007 (16), there have been significant advancements in HCC systemic therapies, opening up new avenues for HCC treatment. Ramucirumab has demonstrated notable efficacy in patients who have failed Sorafenib therapy (17), and Apatinib has shown certain treatment effects in chemotherapy-resistant liver cancer patients. With further research, systemic treatment strategies involving combinations of multiple immune checkpoint blockers and vascular endothelial growth factor inhibitors have been developed. The combination of Nivolumab and Ipilimumab has shown remarkable therapeutic effects in some patients (18). These studies have provided liver cancer patients with more options for systemic treatment and significantly improved patient prognosis. As systemic therapies continue to advance, the combination of surgical and systemic treatments can significantly enhance the effectiveness of liver cancer management. The research focus lies in the systemic treatment before and after surgical procedures. Postoperative systemic therapy, aimed at inhibiting micrometastases and potential residual tumor cells, aims to reduce the recurrence rate and improve long-term prognosis for patients (19). Preoperative systemic treatment aims to convert unresectable liver cancer or liver cancer that does not meet transplantation criteria into resectable tumors, or for resectable liver cancer, to improve its postoperative efficacy (20). However, there is limited research comparing the efficacy of preoperative and postoperative systemic treatments for liver cancer patients, and the comparative effectiveness of the two approaches remains unclear. The SEER database provided available information, and we selected 1918 patients with a well-defined sequence of surgical and systemic treatments. They were divided into two groups: patients who underwent systemic treatment before surgery, and patients who underwent surgery first and received systemic treatment afterwards. A comparative analysis of the efficacy of preoperative and postoperative systemic treatments was conducted.

In this retrospective study, we observed that HCC patients who received systemic therapy before surgery achieved better survival benefits compared to those who received systemic therapy after surgery. This finding is in contrast to previous research (21), which might be attributed to variations in systemic treatment approaches. The research (21), published in 2009, primarily involved traditional cytotoxic chemotherapy as systemic therapy, whereas our study utilized data from the SEER database spanning 2004 to 2015, where patients likely received more tyrosine kinase inhibitors (TKIs) and immunotherapy as systemic treatments following FDA approval of new systemic treatment methods in 2007. We observed a significantly larger number of liver transplant recipients in the preoperative systemic treatment group compared to the postoperative systemic treatment group. Numerous studies have shown that liver transplant can provide liver cancer patients with longer OS and CSS compared to liver resection (22–24). Additionally, liver transplant recipients have a 30% lower recurrence rate than liver resection patients (25). This is because liver transplant not only removes the tumor but also addresses the underlying liver cirrhosis, which is a major risk factor for tumor recurrence (26). To eliminate the impact of surgical approach on the survival rates of the two groups, we conducted PSM. After PSM, the distribution of liver transplant and liver resection in both groups became comparable, and we still reached the same conclusion. This might be attributed to the fact that preoperative systemic treatment can downstage liver cancer patients, making the tumor more amenable to curative treatment, thus providing a better chance of complete resection. Previous research has shown that preoperative systemic treatment for early-stage liver cancer can achieve pathological complete response in 29% of cases (10). In our study, there were significantly more AJCC I+II stage patients than AJCC III+IV stage patients, which could also contribute to the aforementioned results. To explore the differences between different subgroups and the relationship with variables, we conducted further subgroup analyses, including AJCC staging, age, gender, tumor size, and surgical approach. The subgroup analysis revealed that the postoperative systemic therapy group had higher survival risks compared to the preoperative systemic therapy group in different subgroups. This conclusion was drawn without considering PSM, further enhancing the credibility and generalizability of our study findings.

This study incorporated a comprehensive set of variables, representing most prognostic factors that could potentially influence the outcomes of liver cancer patients undergoing surgery combined with systemic therapy, and conducted multiple regression analyses. Consistent findings were observed, both before and after PSM, indicating that compared to preoperative systemic therapy, patients undergoing postoperative systemic therapy have a worse long-term prognosis. To control for other potential factors that may influence OS and CSS, we performed a multivariable regression analysis by gradually adding adjusting variables. We categorized the potential influencing factors into three groups based on the principle of homogeneity: demographic data, including age, gender, race, and marital status; basic tumor characteristics, including the year of diagnosis, AJCC stage, AJCC N stage, AJCC T stage, AJCC M stage, tumor size, and number of tumors; and treatment modalities, including chemotherapy, radiotherapy, and surgical approach. In the univariate analysis, after adjusting for demographic data, adjusting for demographic + basic tumor characteristics, adjusting for all influencing factors, and univariate analysis after PSM, we observed a decreasing trend in hazard ratio (HR) values. This suggests that the adverse impact of postoperative systemic therapy on both overall mortality and cancer-specific mortality, relative to preoperative systemic therapy, is gradually weakening. However, all five groups’ HR values are still greater than 1, indicating that the results are very robust. Previous animal experiments have shown that preoperative systemic therapy is significantly more effective than postoperative systemic therapy (27), which is consistent with our research findings. However, these studies were conducted using a mouse model of breast cancer. Currently, there is a lack of sufficient animal model research specific to HCC in this regard. Evaluating the efficacy of preoperative systemic therapy and postoperative systemic therapy in terms of prolonging patient survival may become an important topic for future research.

This study inevitably has some limitations. Firstly, it is a retrospective study, and inevitably, there may be selection bias. Although we conducted PSM to balance the baseline data of the two groups of patients and achieve comparability, this bias cannot be completely eliminated. In a cohort study, immortal time bias may arise when follow-up includes a period during which participants in the exposed group cannot experience the outcome (28). In the SEER database, survival time is calculated from the time of diagnosis, it seems like that patients must survive sufficiently long to receive treatment, making them “immortal” prior to exposure. But in this study, both groups of preoperative and postoperative systemic treatments have survival time calculated from the time of diagnosis, there is no situation where participants in the exposed group cannot experience the outcome. According to the experience presented in the article (29), calculating survival time from the time of diagnosis is considered a more reasonable and recommended approach. However, the reasons for patients choosing treatment approach are unclear because such information is lacking in the SEER database. This could introduce a bias, as patients’ choice of treatment approach is often related to the stage of their disease progression and risk situation. Secondly, the SEER database primarily includes small molecule targeted drugs and immune checkpoint inhibitors or their combination as systemic treatment. However, specific systemic treatment regimens were not provided, making it challenging to further assess the impact of specific systemic treatment drugs on patient prognosis. Additionally, variables such as AFP levels, liver function status, and liver fibrosis scores are incomplete or missing in the SEER database, and we did not include these factors in the analysis, even though they are essential indicators affecting long-term postoperative survival in liver cancer patients. Nevertheless, our study considered most of the factors that could potentially influence patient prognosis and demonstrated that preoperative systemic treatment offers better survival benefits compared to postoperative systemic treatment. To confirm the validity of the conclusions drawn from the current analysis, prospective studies are necessary. In the near future, several large Phase III clinical trials involving preoperative and postoperative systemic treatment for liver cancer will publish their results, providing valuable clinical decision-making references for liver cancer treatment.

## 5 Conclusion

Based on the SEER database, this study demonstrates that patients with liver cancer who received neoadjuvant systemic therapy experienced significantly better survival outcomes compared to those who received adjuvant therapy after surgery. According to subgroup analysis, it is further deduced that HCC patients aged between 45-75 years, with AJCC stages I to III, and tumor sizes larger than 2cm, exhibited better survival benefits with preoperative systemic treatment compared to the postoperative systemic treatment group.

## Data availability statement

Publicly available datasets were analyzed in this study. This data can be found here: https://seer.cancer.gov.

## Ethics statement

Ethical approval was not required for the study involving humans in accordance with the local legislation and institutional requirements. Written informed consent to participate in this study was not required from the participants or the participants’ legal guardians/next of kin in accordance with the national legislation and the institutional requirements.

## Author contributions

YL: Writing – original draft, Software, Methodology, Formal analysis, Data curation, Conceptualization. SS: Writing – original draft, Formal analysis, Conceptualization. ZC: Writing – original draft, Formal analysis, Data curation. CL: Writing – original draft, Formal analysis, Data curation. LC: Writing – original draft, Formal analysis, Data curation. RZ: Writing – review & editing, Supervision, Project administration, Methodology.
