# Supplementary figures and images for: Comparison of outcomes between preoperative and postoperative systemic treatment in patients with hepatocellular carcinoma: a SEER database-based study
Source: Front Oncol. 2024 Mar 19;14:1324392. doi: 10.3389/fonc.2024.1324392 (PMC10985153; doi:10.3389/fonc.2024.1324392)

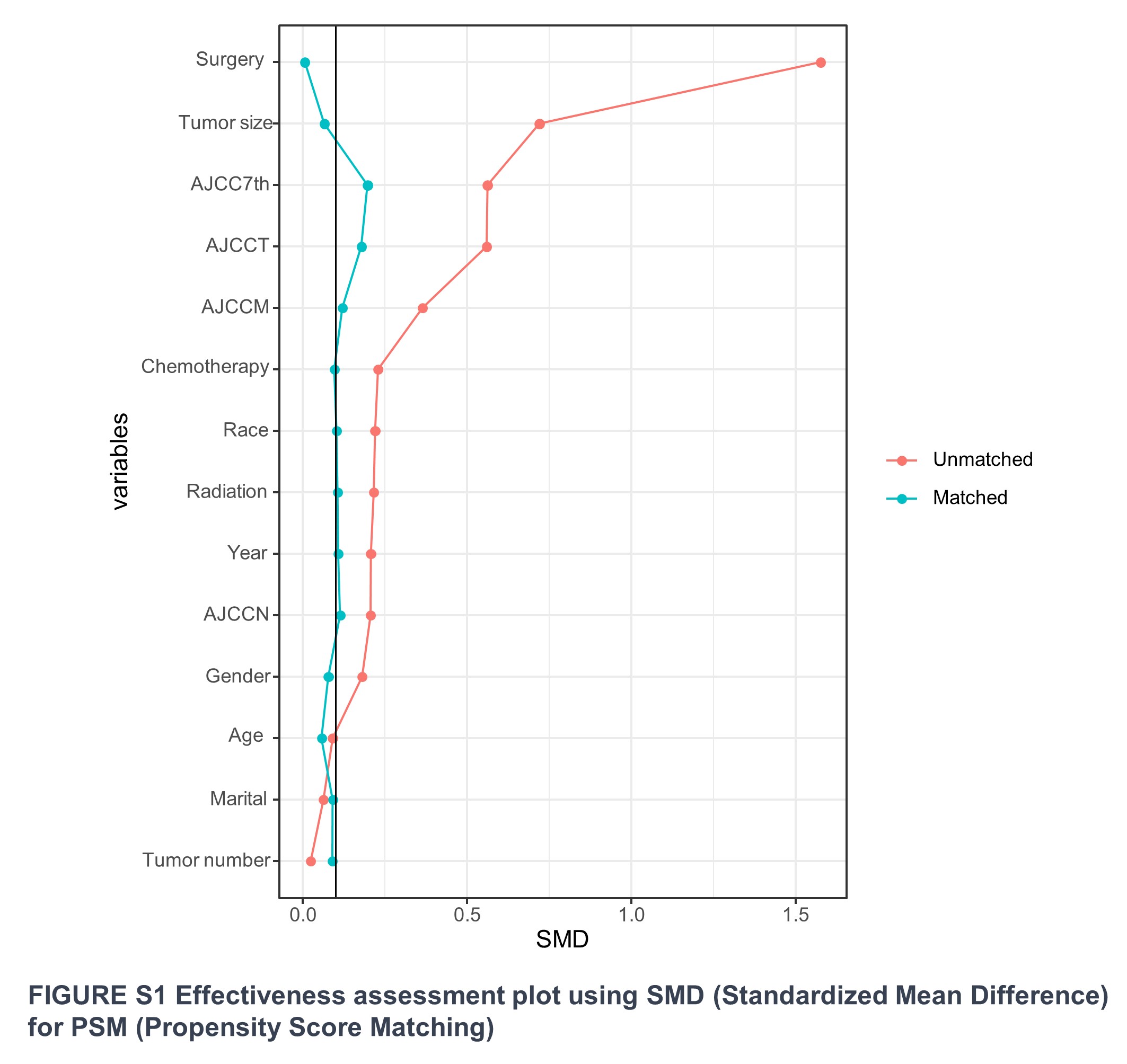

Supplement: Supplementary file 1 [file Image_1.jpeg]
